# Circular RNAs in Cancer: emerging functions in hallmarks, stemness, resistance and roles as potential biomarkers

**DOI:** 10.1186/s12943-019-1002-6

**Published:** 2019-04-18

**Authors:** Min Su, Yuhang Xiao, Junliang Ma, Yanyan Tang, Bo Tian, Yuqin Zhang, Xu Li, Zhining Wu, Desong Yang, Yong Zhou, Hui Wang, Qianjin Liao, Wenxiang Wang

**Affiliations:** 1 Department of the 2nd Department of Thoracic Surgery, Hunan Cancer Hospital and The Affiliated Cancer Hospital of Xiangya School of Medicine, Central South University, Changsha, Hunan 410013 People’s Republic of China; 2 Hunan Key Laboratory of Translational Radiation Oncology, Hunan Cancer Hospital and The Affiliated Cancer Hospital of Xiangya School of Medicine, Central South University, Changsha, China; 3 Department of Pharmacy, Xiangya Hospital of Xiangya School of Medicine, Central South University, Changsha, Hunan 410001 People’s Republic of China; 4 Department of the Central Laboratory, Hunan Cancer Hospital and The Affiliated Cancer Hospital of Xiangya School of Medicine, Central South University, Changsha, Hunan 410013 People’s Republic of China

**Keywords:** CircRNAs, Cancer, Function, Hallmarks, Stemness, Resistance, Biomarker

## Abstract

Circular RNAs (circRNAs) are a class of RNA molecules with closed loops and high stability. CircRNAs are abundantly expressed in eukaryotic organisms and exhibit both location- and step-specificity. In recent years, circRNAs are attracting considerable research attention attributed to their possible contributions to gene regulation through a variety of actions, including sponging microRNAs, interacting with RNA-binding proteins, regulating transcription and splicing, and protein translation. Growing evidence has revealed that circRNAs play critical roles in the development and progression of diseases, especially in cancers. Without doubt, expanding our understanding of circRNAs will enrich knowledge of cancer and provide new opportunities for cancer therapy. In this review, we provide an overview of the characteristics, functions and functional mechanisms of circRNAs. In particular, we summarize current knowledge regarding the functions of circRNAs in the hallmarks, stemness, resistance of cancer, as well as the possibility of circRNAs as biomarkers in cancer.

## Introduction

CircRNAs are a class of single-stranded closed circular RNA molecules that lack 5′-3′ ends and poly (A) tails [1]. Four decades have elapsed since circular RNAs (circRNAs) were first found in plant-based viruses in 1976 [2]. CircRNAs were later found in eukaryotes as an endogenous RNA splicing product in 1979 and in humans following hepatitis delta virus infection in 1986 [3, 4]. However, circRNAs were initially considered as functionless byproducts of aberrant RNA splicing and thus have not garnered sufficient scientific attention. In 2012, Salzman et al. [5] identified the abundance of circRNA species in both normal and mammalian cells and revealed that more than 10% of expressed genes are able to produce circRNAs. In 2013, Hansen et al. [6] and Memczak et al. [7] reported that circular transcripts of cerebellar degeneration-related protein 1 antisense RNA (CDR1as, also known as ciRS-7) can serve as miRNA sponges for miR-7. These works transformed circRNAs into a focal point of scientific research and rising stars in the noncoding RNA field.

In recent years, following the development and application of high-throughput deep RNA sequencing and bioinformatics technology, circRNAs have been found to be widespread in eukaryotic cells and dynamically expressed in various developmental stages and physiological conditions [8, 9]. A large number of researchers have demonstrated that circRNAs are correlated with the pathogenesis of various human diseases, including nervous system disorders [10], cardiovascular disorders [11], Alzheimer’s disease [12], osteoarthritis [13], diabetes [14], silicosis [15] and cancer [16, 17]. In particular, circRNAs have been reported to play critical roles in cancer growth, metastasis, stemness and resistance to therapy [18, 19]. Natural circRNA, which plays an important role in the RNA interaction network, was proven to be extremely abundant, relatively stable, diverse and conserved [8]. Emerging evidence suggests that circRNAs are responsible for complicated functions such as serving as endogenous RNAs to sponge miRNAs, regulating expression of parental genes, modulating alternative splicing, regulating RNA–protein interactions, and acting as scaffolds in the assembly of protein complexes [20, 21]. In this review, we describe the characteristics, functions and functional mechanisms of circRNA. Specifically, we discuss the role of circRNA in the hallmarks, stemness, resistance of cancer, as well as the possibility of circRNAs as biomarkers in cancer.

### Characteristics and biogenesis of circRNAs

According to recent research, circRNAs are typically generated from one to five exons with length between a few hundred to thousands of nucleotides (nt) [22, 23]. There are several important properties of circRNAs generated by back-splicing: (1) circRNAs have a closed ring structure—without either 5′–3′ polarity or a polyadenylated tail—and are thus insusceptible to degradation by exonucleases and much more stable than linear RNA [24]; (2) circRNAs are widely expressed in eukaryotic cells, and more than one million circRNAs exist in human tissues as detected by high-throughput sequencing [25]; (3) circRNAs primarily reside in the cytoplasm, whereas a small number of circRNAs are located in the nucleus [7]; (4) most circRNAs have highly conserved sequences between different species [26]; (5) circRNAs exhibit tissue-specific and dynamic developmental stage-expression patterns [9]; (6) circRNAs play a regulatory role at the level of transcription or posttranscription [7].

**Fig. 1:**
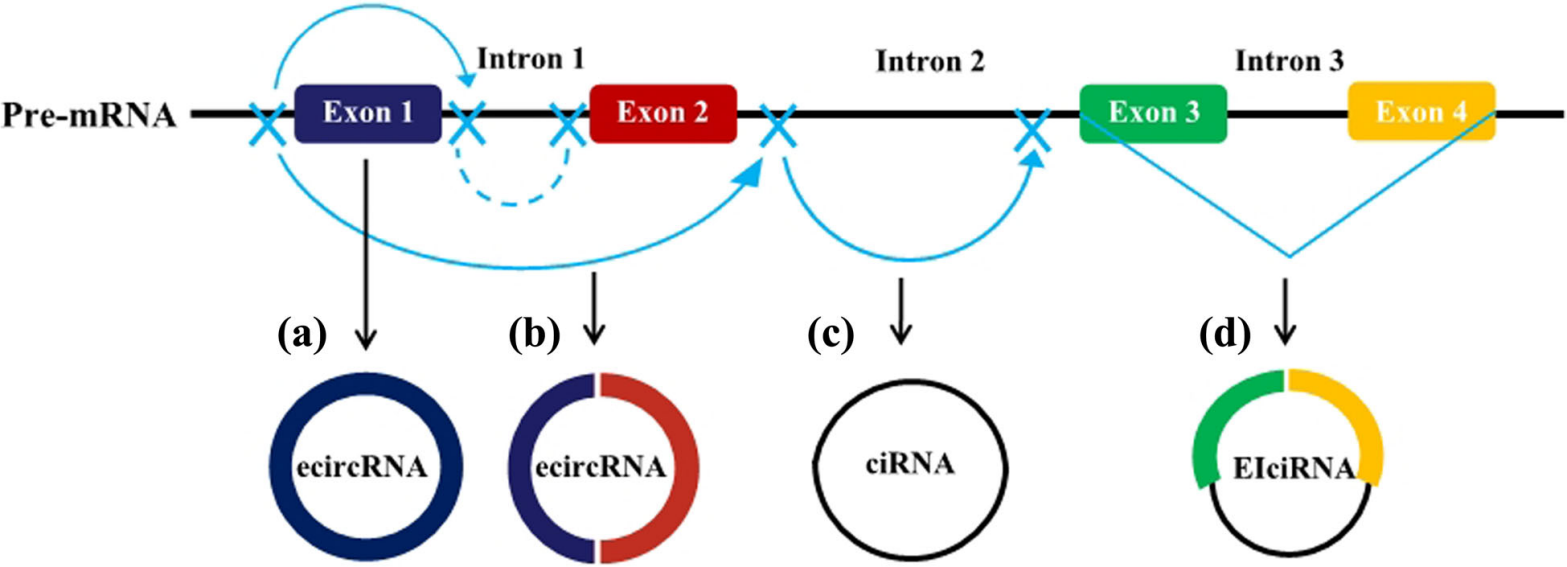
Formation of three types of circRNAs. **a** Exonic circular RNA (ecircRNA) is formed through back-splicing of the 5’splice site (splice donor site) to a 3’splice site (splice acceptor site); (**b**) The intron 1 is removed and bring the 5′ splice site of Exon 2 close to 3′ splice site of Exon 1, to form a ecircRNA that contains multiple exons. Exons can also skip splicing, exon 1 can also link with exon 3; (**c**) Circular intronic RNA (ciRNA) are derived from intron lariats that escape the normal intron debranching and degradation. Reverse complementary sequences of lariat intron excised from pre-mRNA can pair to produce close loop structure termed as ciRNA; (**d**) Exon–intron circRNAs (EIciRNAs) are circularized with introns‘retained’between the exons. Intron 3 retaines with Exon 3 and Exon 4 to form an EIciRNAs

Both circRNAs and linear RNAs are originated from precursor mRNAs (pre-mRNAs), but in contrast to linear RNAs that are generated by classical splicing, circRNAs are usually formed by back-splicing [8]. CircRNAs can be derived from all regions of the genome, including intergenic, intronic, antisense and untranslational regions [7]. There are three major categories of circRNAs base on their origin: exonic circRNAs (ecircRNAs), exon-intron circRNAs (EIciRNAs), and circular intronic RNAs (ciRNAs) [27]. EcircRNAs are derived from exons and account for the main part of identified circRNAs [28]. Two models of ecircRNA formation have been proposed [8]. (1) Iariat-driven circularization model: the introns in a lariat intermediate that consists several exons and introns are removed, followed by the connection between the 3′ splice site of an upstream exon (splice acceptor) and the 5′ splice site of a downstream of exon (splice donor), resulting in the formation of ecircRNAs. (2) Intron pairing-driven circularizing model: a circular structure is formed by base-paring between reverse complementary sequences (such as Alu repeats, which are short DNA stretches initially characterized by the action of the Arthrobacterluteus restriction endonuclease) across exon-flanking introns. Intron paring place the splice sites close to each other, followed by back-splicing of pre-mRNAs and exon circularization. Unlike ecircRNAs, EIciRNAs retain the introns that are not spliced out completely [29]. Pre-mRNAs that contain flanking Alu complementary pairs or flanking complementary sequence pairs other than Alu could facilitate the production of EIciRNAs [29]. In addition, ciRNAs are derived from intron lariats that escape the normal intron debranching and degradation [30]. The formation of ciRNAs are dependent on the presence of a 7 nt GU-rich sequence near the 5′ splicing site and a 11 nt C-rich motifs near the 3′ branch point site. Up to today, several sequence features have been indicated to influence the biosynthesis of circRNA, such as length of intron and exon, repetitive sequences and RNA-binding proteins (RBPs) [31, 32]. The RBPs that include muscleblind (MBL), quaking (QKI), SR protein, adenosine deaminases that act on RNA (ADAR1), fused in sarcoma (FUS), heterogeneous nuclear ribonucleoprotein (hnRNP), NF90/NF110, heterogeneous nuclearribonucleoprotein L (HNRNPL) and muscleblind (MBL), could positively or negatively regulate the formation of circRNAs [32–35] (Fig. 1).

### Functional mechanisms of circRNAs

#### Act as miRNA sponges

Multiple lines of evidence have proven that some circRNAs are rich in miRNA response elements (MREs) and may serve as miRNA sponges. MiRNAs are small, noncoding RNAs with approximately 22 nt lengths that play an important role in posttranscriptional gene expression through binding to specific target sites within the mRNA 3′-untranslated region (3′-UTR), leading to decreased mRNA stability and suppression of translation [36, 37]. CircRNAs may regulate gene expression through binding to and releasing miRNAs from their downstream target genes [38, 39]. In comparison with other miRNA sponges, some circRNAs exhibit a superior ability to bind with miRNAs and have been referred to as “super sponge” [21]. The best example is CDR1as, which harbors more than 70 selectively conserved miR-7 binding sites [40]. In addition, there are a large number of examples of circRNAs able to act as miRNA sponges, including circRNA ZNF609 [41], circ-SRY [42], mm9_circ_012559 [43], circDOCK1 [44], and many others.

#### Interact with RNA binding proteins

In addition to acting as miRNA sponges, some circRNAs that harbor binding sites for RNA-binding proteins may serve as protein sponges or decoys and thus regulate gene expression. For instance, the circRNA originating from the PABPN1 locus (circ-PABPN1) binds to human antigen R/ELAV-like protein 1 (HuR) and prevents HuR from binding to PABPN1 mRNA, subsequently suppressing PABPN1 translation [45]. The other examples, including circ-Foxo3 [46] and circ-Mbl [31], primarily interact with RNA binding proteins.

#### Regulate transcription or splicing

Some circRNAs have been demonstrated to regulate gene transcription through combining with RNA polymerase II complex and translating related proteins [47]. For example, circ-EIF3J and circ-PAIP2 were found to interact with the U1 snRNPs and RNA polymerase II in the promoter region of the host gene to realize enhanced transcription of their parental genes, such as PAIP2 and EIF3J [29].

Studies have also suggested that circRNAs can contribute to the regulation of selective splicing. A study by Ashwal-Fluss et al. [31] showed that circMbl is derived from the circularization of the second exon of the splicing factor muscleblind (MBL) and could compete with linear MBL mRNA for selective splicing. Notably, due to the presence of functional circMbl binding sites in the MBL protein, MBL could interact with circMbl and promote circMbl production. Thus, circMbl negatively affects canonical splicing and decreases the production of the parental mRNA.

#### Translate proteins

Because of lacking 5′-3′ polarity and polyadenylated tails, as well as internal ribosome entry sites (IRES), circRNAs were initially defined as a distinct class of endogenous noncoding RNA that could not translate proteins [48, 49]. However, convincing evidence has shown that some circRNAs possess translational ability. To this point, at least four circRNA molecules have been proven to be translatable. Legnini I. et al. [50] revealed that circ-ZNF609 contains an open reading frame (ORF) and could be translated into a protein in murine myoblasts when driven by IRES. Additionally, circ-SHPRH [51] and circ-FBXW7 [52], as well as proteins encoded by them, are found to be abundantly expressed in normal human brains but downregulated in glioma. Both of the circRNAs have an ORF driven by the IRES to translate a functional protein. Analogously, Pamudurti N. R. et al. [53] found that circMbl can also translate protein in a cap-independent manner.

#### Regulate epigenetic alterations

**Fig. 2:**
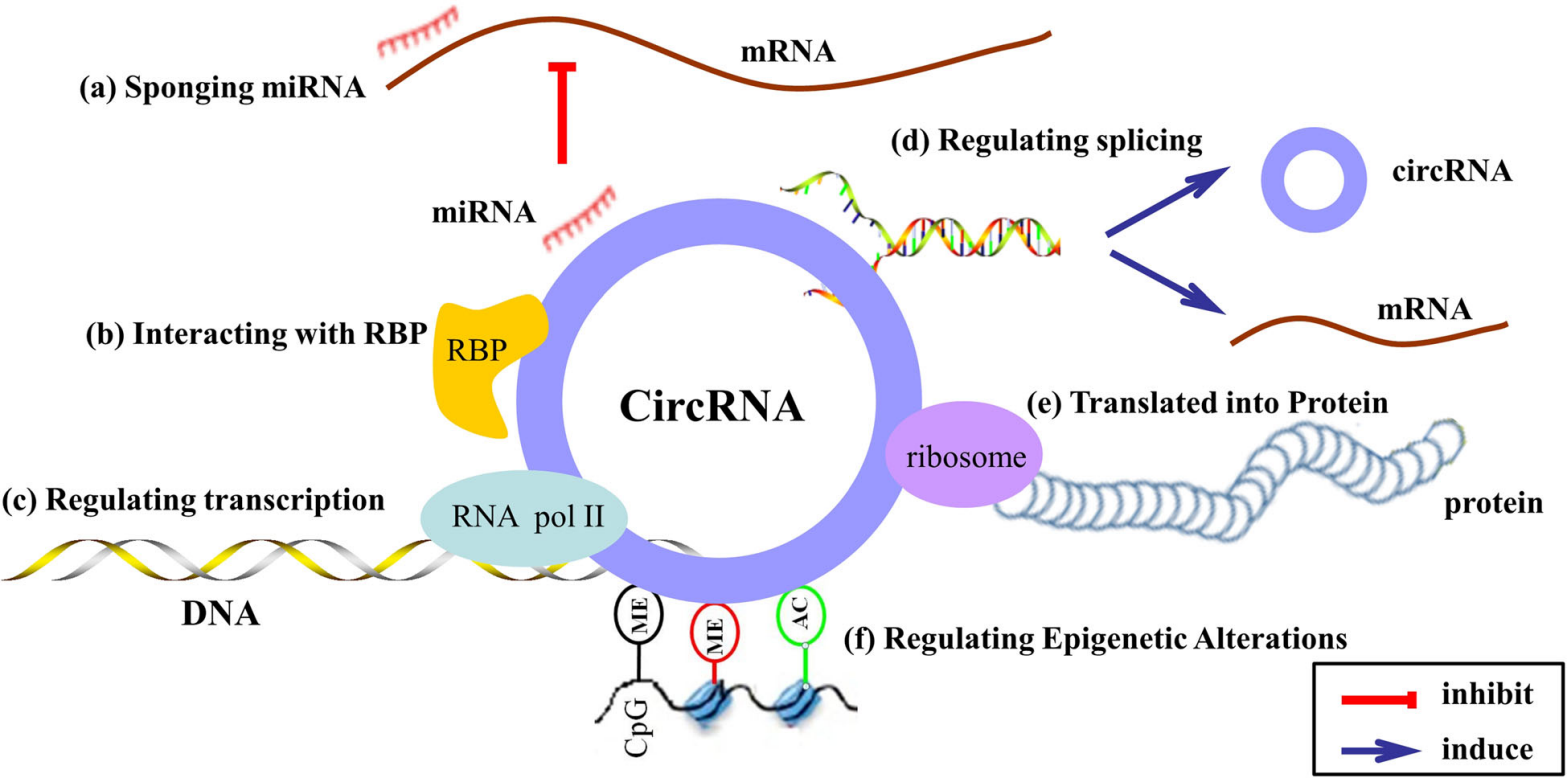
The functional mechanisms of CircRNAs. **a** Acting as miRNA sponge; (**b**) Binding with RNA binding protein (RBP); (**c**) Regulating transcription; (**d**) Regulating splicing; (**e**) Translated into Protein; (**f**) Regulating epigenetic alterations

Aberrant DNA methylation and histone modifications that associated with epigenetic gene expression are frequently found in cancer [54, 55]. Some circRNAs have been found to regulate these epigenetic alterations. Chen et al. [56] reported that circFECR1 induced extensive CpG DNA demethylation in the promoter of FLI1 and thus epigenetically activated FLI1. CircFECR1 was demonstrated to downregulate the transcription of DNMT1, a critical methyltransferase required for the maintenance of DNA methylation, through binding to the DNMT1 promoter. In addition, circFECR1 could recruit TET1 DNA demethylase to the FLI1 promoter and induce DNA demethylation. Enhancer of zeste homolog 2 (EZH2) is a subunit of polycomb-repressive complex 2 (PRC2), which functions as a H3K27 methyltransferase and regulates histone methylation [57, 58]. Several circRNAs have been reported to regulate EZH2 expression through acting as miRNA sponges, subsequently regulate histone methylation indirectly. For example, circBCRC4 is able to promote the expression of EZH2 by binding with miR-101 [59], hsa_circ_0020123 is able to upregulate EZH2 and ZEB1 through sponging miR-144 [60], hsa_circ_0071589 can regulate the miR-600/EZH2 signaling [61] (Fig. 2).

### CircRNAs regulate the hallmarks of cancer

**Fig. 3:**
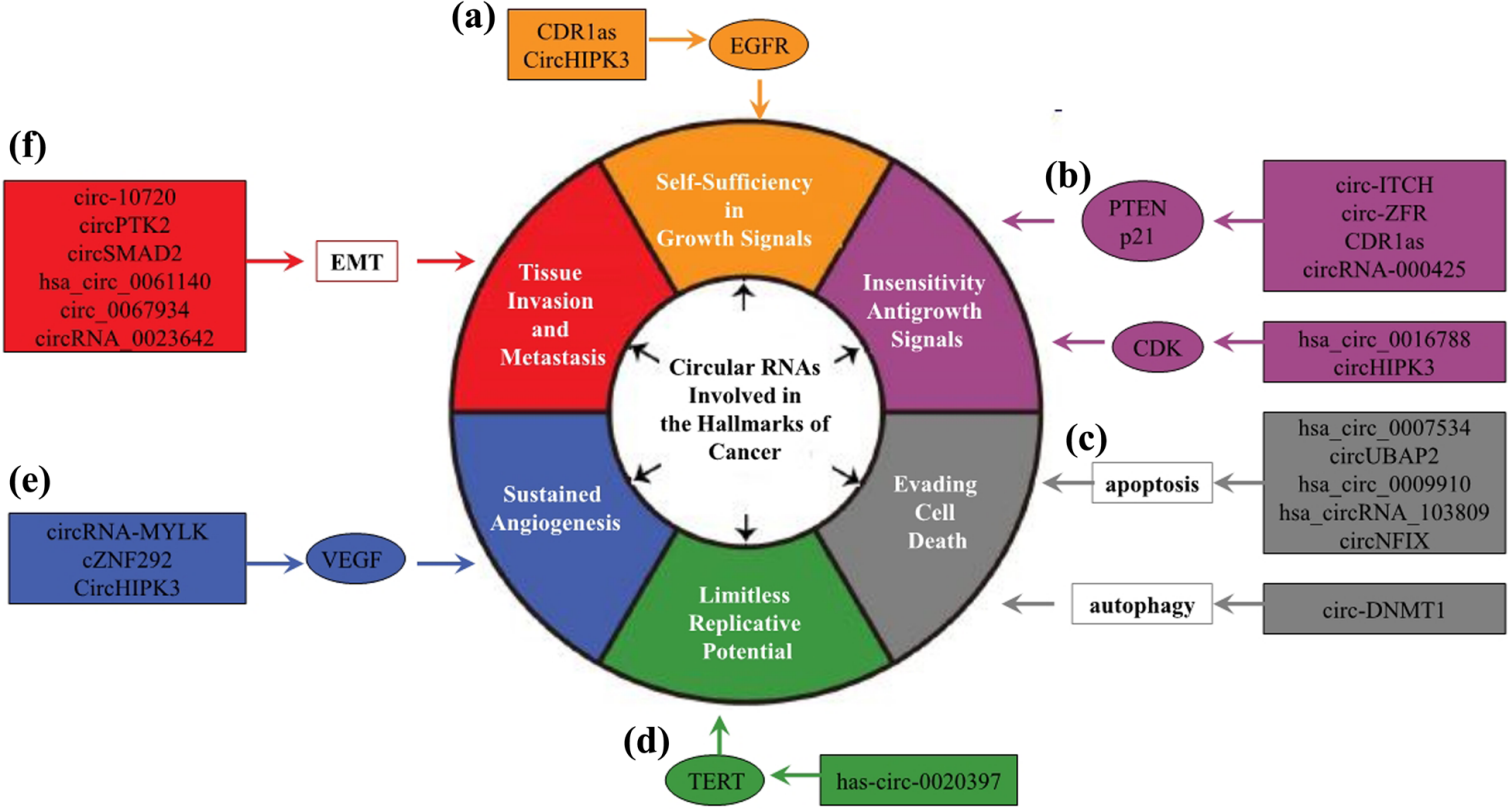
CircRNAs involved in the hallmarks of cancer. **a** CDR1as and circHIPK3 active cell proliferative through regulating EGFR; (**b**) Circ-ITCH et al. promote cancer cells evading antigrowth signals by preventing expression or activation of tumor suppressors, such as PTEN and CDK; (**c**) Hsa_circ_0007534 et al. promote cancer cells evading cell death via regulating cellular apoptosis or autophagy; (**d**) Has-circ-0020397 limits replicative potential of cancer cell trough regulating TERT; (**e**) CircRNA-MYLK et al. sustain angiogenesis trough regulating VEGF; (**f**) Dirc-10,720 et al. regulate the process of EMT and thus tissue invasion and metastasis of cancer

In 2000, Hanahan and Weinberg proposed six hallmarks of cancer that result in the progressive conversion of normal cells into cancerous cells [62]. Most and perhaps all types of human cancer shared these acquired capabilities, including self-sufficiency in growth signals, evasion of antigrowth signals, resistance to cell death, limitless replicative potential, sustained angiogenesis, tissue invasion and metastasis. In recent years, some circRNAs have been shown to be involved in these properties of cancer (Fig. 3 and Table 1).

**Table 1:** CircRNAs involved in the hallmarks of cancer

Function	CircRNA	Cancer type	expression	Associated clinical features	Associated cell process	Targets	Ref.
Sustaining growth signaling	CDR1as	NSCLC	up	TNM stage, lymph nodes metastasis and survival time	promote cell vitalities and growth, inhibit apoptosis and cell cycle arrest	miR-7/EGFR, CCNE1, PIK3CD.	[72]
	CDR1as	CRC	up	advanced tumor stage, tumor depth, and survival time	–	miR-7/EGFR, RAF1	[73]
	CDR1as	HCC	down	–	promote cell growth, adhesion, and inhibit migration	miR-7/EGFR	[74]
	circHIPK3	CRC	up	metastasis, clinical stage and survival time	inhibite cell proliferation, migration, invasion, and induce apoptosis	miR-7/EGFR, FAK, IGF1R, YY1	[75]
	hsa_circ_0046701	glioma	up	–	promote cell proliferation and invasion	miR-142-3p/ITGB8	[80]
	circ-FBXW7	glioblastoma	down	survival time	inhibit cell proliferation and cell cycle acceleration	FBXW7-185aa/c-Myc	[52]
	Circ-Amotl1	–	up	–	promote cell proliferation	c-myc	[84]
Evading growth inhibitors	circ-ITCH	Bca	down	histological grade and survival time	inhibite cell proliferation, migration, invasio, induce G1/S cell cycle arrest and apoptosis	miR-17, miR-224/p21, PTEN axis	[90]
	circ-ZFR	GC	down	–	inhibit cell propagation, cell cycle and promote apoptosis	miR-130a, miR-107/PTEN	[91]
	CDR1as	GC	up	lymph nodes metastasis and survival time	promote cell proliferation, migration and inhibit apoptosis	miR-7/PTEN/PI3K/AKT	[92]
	circRNA-000425	GC	down	–	inhibit cell growth	miR-17,miR-106/p21, BIM	[93]
	Circ100284	–	up	–	promote cell cycle acceleration	miR-217/EZH2/cyclin D1, CDK4	[97]
	hsa_circ_0016788	HCC	up	–	promote cell proliferation, invasion and inhibit apoptosis	miR-486/CDK4 axis	[98]
	circHIPK3	gallbladder cancer	up	–	promote cell survival and proliferation, inhibit cell apoptosis	miR-124/ROCK1, CDK6	[99]
Resisting apoptosis	hsa_circ_0007534	CRC	up	tumor stage and lymph node metastasis	ptomote cell proliferation and inhibit apoptosis	Bcl-2, Bax	[104]
	circUBAP2	osteosarcoma	up	tumor progression and prognosis	promote cell growth and inhibit apoptosis	miR-143/Bcl-2	[105]
	hsa_circ_0009910	osteosarcoma	up	–	promote cell proliferation inhibition, inhibit cell cycle arrest, and inhibit apoptosis	miR-449a/IL6R/Bcl-2/Bax	[106]
	hsa_circRNA_103809	CRC	down	–	promote apoptosis	miR-532-3p/FOXO4 axis	[107]
	circNFIX	glima	up	–	promote cell propagation, migration and inhibit apoptosis	miR-34a-5p/NOTCH1	[108]
	circ-DNMT1	BC	up	–	inhibit autophagy, promote cell proliferation and survival	p53, AUF1	[110]
Uncontrolled replicative immortality	has-circ-0020397	CRC	up	–	promote cell viability and inhibit apoptosis	mir-138/TERT, PD-L1	[114]
Promoting angiogenesis	circRNA-MYLK	Bca	up	pathological stage, T and N classifications and survival time	promote cell growth, angiogenesis and metastasis	miR-29a/VEGFA/VEGFR2	[118]
	cZNF292	Glima	up	–	promote cell proliferation, tube formation and angiogenic potential	VEGF-A, EGF, TGF-β1	[122]
	circHIPK3	Bca	down	tumor grade, invasion, lymph node metastasis	inhibit migration, invasion, and angiogenesis	miR-558/HPSE/VEGF	[119]
Activating invasion and metastasis	circ-10,720	HCC	up	Tumor metastasis and survival time	promote cell proliferation, migration, invasion and EMT	Vimentin	[129]
	circPTK2	NSCLC	down	–	inhibit cell invasion and EMT	miR-429, miR-200b-3p/TIF1y	[132]
	circSMAD2	HCC	down	–	inhibit cell migration, invasion, and EMT	miR-629	[137]
	hsa_circ_0061140	ovarian cancer	up	–	promote cell proliferation, migration, invasion and EMT	miR-370/FOXM1	[141]
	circ_0067934	NSCLC	up	TNM stage, lymph node statu, distant metastasis and survival time	promote cell proliferation, migration, invasion and EMT	N-cadherin, vimentin snail, and E-cadherin	[142]
	circRNA_0023642	GC	up	–	pormote cell proliferation, migration, invasion and EMT	N-cadherin, vimentin snail, and E-cadherin	[143]`

#### Self-sufficiency in growth signals

Normal cells acquire self-sufficiency in growth signals to change into an active proliferative state [62]. The mitogenic growth signals are transmitted into the cell interior by binding to the transmembrane receptors. Cancer cells could produce dysregulated growth factors and/or the corresponding receptor molecules themselves to lead to an autocrine stimulation.

Epidermal growth factor receptor (EGFR), highly expressed in a variety of solid tumors, is a critical molecular signal that can trigger an intracellular transduction cascade of growth factors and regulate cell growth [63, 64]. A variety of studies have shown that EGFR is a target of miR-7, which is a tumor suppressor regulating various biological processes [65, 66]. One of the most well-known circRNAs, CDR1as, harbors more than 70 selectively conserved miR-7 target sites, thus acting as a sponge of miR-7 [6, 7]. CDR1as, predominantly found in human brain, is approximately 1500 nt in length [67]. CDR1as has been reported to be involved in pulmonary fibrosis, myocardial infarction, insulin secretion, neuropsychiatric disorders, and cancer [68–71]. Zhang et al. [72] reported that CDR1as was highly expressed in non-small cell lung cancer (NSCLC) tissues, correlated with TNM stage, lymph node metastasis and survival time, and acted as an independent prognostic factor for the NSCLC patients. Knockdown of CDR1as promotes cell vitality and growth through induction of cell apoptosis and cell cycle arrest in G1/S phase. Mechanical assays revealed that CDR1as functioned as a miR-7 sponge to increase the expression levels of miR-7 targeting proto-oncogenes (EGFR, CCNE1 and PIK3CD). In addition, CDR1as was also markedly overexpressed in colorectal cancer (CRC) tissues and correlated with advanced tumor stage, lymph node involvement, distant metastasis and poor patient survival of CRC patients [73]. Overexpression of CDR1as led to blocking of the tumor suppressive effects of miR-7 and resulted in a more aggressive oncogenic phenotype. Overexpression of CDR1as induced inhibition of miR-7 and subsequent activation of miR-7 target oncogenes EGFR and RAF1. However, in another study, CDR1as expression was shown to be downregulated in hepatocellular carcinoma (HCC) tissues and cell lines [74]. The expression profiles differ between NSCLC, CRC and HCC, possibly due to the tissue-specificity of circRNAs. Ectopic expression of CDR1as could increase cell growth and adhesion while inhibiting the migration of HCC cells. CDR1as exhibited these functions through regulating the expression of EGFR. Another circRNA that regulates EGFR is circHIPK3 (hsa_circ_0000284), which originates from the HIPK3 gene exon 2, with the length of 1099 nt [75, 76]. CircHIPK3 is a particularly abundant circRNA that has been proposed to be involved in tumorigenesis [76, 77]. It was shown to be markedly overexpressed in CRC tissues and cell lines and positively associated with advanced clinical stage and poor survival of CRC patients [75]. CircHIPK3 knockdown significantly inhibited CRC cell proliferation while inducing cell apoptosis. Interestingly, miR-7 was identified and confirmed to be the only miRNA that directly interacts with circHIPK3 [75]. Furthermore, overexpression of circHIPK3 effectively reversed miR-7-induced attenuation of CRC cell progression through upregulating the expression of several key miR-7 target genes, including EGFR, IGF1R, FAK and YY1.

However, EGFR is not the only growth signal regulated by circRNAs. Integrins are transmembrane receptors and mediators of the interactions between cells and the extracellular matrix (ECM) [78]. Integrin-mediated interactions are required for the cytoskeletal organization, attachment, survival, proliferation, differentiation and migration of cells. Following binding to specific moieties of the ECM, the integrin receptors are able to transduce signals into the cell that mediate cell behavior. Integrin subunit beta 8 (ITGB8) is an important member of the integrin family [79]. A recently study showed that hsa_circ_0046701, which was highly expressed in glioma tissues and cell lines, was able to promote cell proliferation and invasion through regulating ITGB8 expression by sponging miR-142-3p [80].

C-myc, an important transcription factor, acts as an oncogene to regulate various cellular processes including cell proliferation, differentiation, and apoptosis [81, 82]. It has been reported that c-myc can regulate up to 15% of gene expression [83]. Yang and coworkers [52]conducted the RNA-expression profiling from glioblastoma and matched noncancerous tissues and characterized the circular form of the FBXW7 gene, circ-FBXW7. Circ-FBXW7 was downregulated in glioblastoma tissues and correlated with overall survival of glioblastoma patients. The spanning junction open reading frame in circ-FBXW7 driven by internal ribosome entry site encoded a novel 185-amino acid protein, which was termed as FBXW7-185aa. FBXW7-185aa, but not circ-FBXW7, could function as a tumor suppressor to induce cell cycle arrest and inhibit proliferation in glioma cells through reducing the half-life of c-myc. FBXW7-185aa was shown to directly interact with de-ubiquitinating enzyme USP28, and thus antagonize the USP28-induced de-ubiquitination of c-Myc and increase c-Myc ubiquitination. Another circRNA that could regulate c-myc is circRNA derived from angiomotin-like1 (circ-Amotl1) [84]. Circ-Amotl1 was highly expressed in breast cancer (BC) tissues and cell lines. Knockdown of circ-Amotl1 promoted cell proliferation and inhibited apoptosis of BC cells. In addition, circ-Amotl1 was demonstrated to bind to c-myc, translocate into nucleus and prevented degradation of c-myc. Ectopic expression of circ-Amotl1 enhanced the binding affinity of c-myc to the promoters of a number of its targets, including HIF-1α, Cdc25a, ELK-1, and JUN. However, how circ-Amotl1 prevents c-myc degradation is not clear and awaits further investigation.

#### Insensitivity antigrowth signals

There are multiple antiproliferative signals in normal cells that operate to maintain cells in a quiescent state [62]. These signals can block cell proliferation through arresting the cell cycle [85]. However, cancer cells can evade antigrowth signals by preventing expression or activation of tumor suppressors.

Phosphatase and tensin homolog (PTEN), deleted from chromosome ten and mutated at high frequency in a variety of cancers, can contribute to the G0-G1 cell cycle regulation and additional cellular functional activities [86, 87]. Cyclin-dependent kinase (CDK) inhibitor p21 is a cell cycle suppressing protein, and overexpression of p21 acts to suppress cell growth [88, 89]. Circ-ITCH, the circularized product from several exons of itchy E3 ubiquitin protein ligase homolog (ITCH), has been reported to be downregulated in bladder cancer (BCa)tissues and correlated with the histological grade and shortened survival of BCa patients [90]. It was also downregulated in BCa cell lines, and the enforced expression of circ-ITCH inhibited cell proliferation, migration, invasion and metastasis. Mechanical assays demonstrated that circ-ITCH could directly sponge miR-17 and miR-224 and lead to increased expression of their target genes, PTEN and p21. Another circRNA, circ-ZFR, was shown to be downregulated in gastric cancer (GC); circ-ZFR regulated GC progression by directly binding with miR-130a/miR-107, and further regulated the expression of PTEN, which is a target of these miRNAs [91]. In addition, CDR1as was overexpressed in GC tissues and correlated with poor survival [92]. Ectopic expression of CDR1as increased expression of PTEN through sponging of miR-7 and subsequently activated the PTEN/PI3K/AKT pathway. Liu and colleagues [93] identified circRNA-000425 as a novel inhibitory target of Yes-associated protein 1 (YAP1), an transcriptional coactivator factor that acts as an oncogene associated with cancer malignancy in several cancer types [94, 95]. YAP1 could suppress the expression of circRNA-000425 through binding to the promoter of HNRNPH1, which codes for circRNA-000425. CircRNA-000425 was identified as a sponge of miR-17/miR-106b and indirectly modulated their targets, such as p21 and BIM, thus suppressing GC cell growth.

In addition to these well-known tumor suppressors, some circRNAs could also regulate tumor growth by regulating cell cycle mediators, such as Cyclin D1, a well-known regulator of the cell cycle that promotes the transition from G1 to S phase by activating CDK4 or CDK6 [96]. Xue and coworkers [97] performed a circRNA microarray to analyze the variability of circRNAs in arsenite-treated HaCaT (As-HaCaT) cells and in arsenite-transformed cells compared to normal HaCaT cells and identify cirRNAs that involved in arsenite-induced acceleration of the cell cycle. Circ100284 was greatest up-regulated in As-HaCaT cells and showed the most change following arsenite treatment. Knockdown of circ100284 inhibited G1/S transition in As-HaCaT cells. Circ100284 was demonstrated to be involved in the arsenite-promoted cell cycle through regulation of EZH2 via sponging miR-217. EZH2 subsequently bind to the promoter of CCND1, the host gene of cyclin D1. Knockdown of EZH2 suppressed the expression of cyclin D1 and CDK4. Although EZH2 frequently acts through methylation of H3K27 as discussed above, here, it functions through the methylase-independent pathway. These results suggested that, in HaCaT cells, circ100284 was induced by arsenite treatment and acted as a sponge for miR-217 to up-regulate EZH2, which, in turn, increased the expression of cyclin D1 and CDK4, and thus lead to cell cycle acceleration and malignant transformation. In another study, Guan et al. [98] performed circRNA microarray analysis in HCC tissue and identified a highly expressed circRNA, hsa_circ_0016788. Silencing of hsa_circ_0016788 inhibited proliferation and promoted apoptosis of HCC cells through regulation of the miR-486/CDK4 axis. CircHIPK3 was shown to be overexpressed in human gallbladder cancer cells [99]. Silencing of circHIPK3 decreased the proliferative and survival capacities, induced apoptosis of gallbladder cancer cells through sponging the tumor-suppressive miR-124, and increased expression of ROCK1 and CDK6, which are miR-124 targets [100, 101].

#### Evading apoptosis

Apart from the rate of cell proliferation, the rate of cell attrition also determines the populations of cells [62]. Apoptosis is the major mechanism leading to this attrition. The other two pathways related to cell attrition are autophagy and necrosis [85]. Cancer cells acquired the ability to evade these signals.

It is well known that B-cell lymphoma-2 (Bcl-2) is an important anti-apoptotic molecule that protects cells from apoptosis, while BCL2-associated X protein (Bax) is a proapoptotic gene [59, 102]. Thus, the ratio of Bcl-2/Bax is a profound indicator of cell survival [103]. Hsa_circ_0007534 was significantly overexpressed in CRC tissues and related to tumor stage and lymph node metastasis [104]. Silencing of hsa_circ_0007534 inhibited proliferation while promoting the apoptosis of CRC cells. Moreover, the Bcl-2/Bax ratio was decreased following hsa_circ_0007534 silencing, which demonstrated that hsa_circ_0007534 inhibits CRC cell proliferation, at least partially, by inducing apoptosis. However, how hsa_circ_0007534 regulates the Bcl-2/Bax ratio is so far unknown and further studies will need to unravel the molecular mechanism.

Zhang et al. [105] performed microarray experiments to examine the expression profiles of circRNAs in osteosarcoma tissue and found that circUBAP2 was the most markedly increased circRNA. CircUBAP2 was also significantly overexpressed in osteosarcoma cells. CircUBAP2 knockdown inhibited cell proliferation and promoted cell apoptosis. Mechanistically, circUBAP2 was found to directly bind to and inhibit the expression of miR-143, thus enhancing the expression of the miR-143 target Bcl-2. Another study performed by Deng et al. [106] showed that hsa_circ_0009910 was upregulated in osteosarcoma cells. Circ_0009910 knockdown inhibited cell proliferation and induced cell cycle arrest and apoptosis in osteosarcoma cells. Circ_0009910 was found to directly bind to and function as a sponge of miR-449a, thereby regulating the target gene IL6R as well as the downstream Bcl-2 and Bax.

In addition, there are several additional circRNAs involved in apoptosis. For example, Hsa_circRNA_103809 was downregulated in CRC and could promote apoptosis through the miR-532-3p/FOXO4 axis [107]. CircNFIX was overexpressed in glioma and inhibited apoptosis through regulating NOTCH1 via binding to and sponging miR-34a-5p [108].

Apart from apoptotic roles, there is also a circRNA involved in autophagy. It has been reported that nuclear translocation of p53 could induce cellular autophagy [109]. Circ-Dnmt1, generated from Exons 6 and 7 of the mRNA NM_001130823.1, was found to be upregulated in tissues of BC as well as in eight BC cell lines [110]. Overexpression of circ-Dnmt1 increased cell survival and proliferation of BC cells through stimulating cellular autophagy. In addition, nuclear levels of circ-Dnmt1 were increased in autophagy inducer-treated BC cells, indicating that autophagy could enhance the nuclear translocation of circ-Dnmt1. Circ-Dnmt1 could directly bind with p53, promoting its nuclear translocation.

#### Limitless replicative potential

Replicative potential is limited because of the appearance of two processes termed as senescence or crisis in normal cells [62]. The telomeres that locate at the chromosome ends are critical for this finite replicative potential: they shorten after every cell division, and therefore, the number of cell division cycles is dictated by the length of telomeres [111]. Approximately 85–90% of human cancers overexpress telomerase, which adds telomeric repeats onto the ends of telomeric DNA, suggesting that limitless replicative potential is essential for the development of their malignant growth [112].

Telomerase reverse transcriptase (TERT) is a catalytic subunit of telomerase [113]. Zhang and colleagues [114] reported that hsa_circ_0020397, derived from the DOCK1 gene, was upregulated in CRC cells, promoted their viability, and inhibited apoptosis. By using a common bioinformatic algorithm, the authors predict that an important cancer suppressor, miR-138, possesses multiple binding sites on hsa_circ_0020397. In addition, has_circ_0020397 was demonstrated to inhibit the activity of miR-138, although it did not influence miR-138 expression, and increase the expression of miR-138 target genes including PD-L1 and TERT.

#### Sustained angiogenesis

Tumor size increases when cancer cells grow. However, the size is limited to within 100–200 μm without angiogenesis due to the limited natural diffusion capability of oxygen and nutrients [115]. Angiogenesis is the process induced by tumor cells that forms new blood vessels in order to supply the tumor with oxygen and nutrients and to dispose of tumor metabolic (toxic) wastes.

Vascular endothelial growth factor (VEGF) is believed to be the most potent mediator of crucial regulatory roles in angiogenesis [116, 117]. CircRNA-MYLK is spliced from MYLK gene, with the spliced mature sequence length of 376 nt [118]. It was significantly overexpressed in BC tissues and correlated with the clinical features of BC patients including the pathological stage, T and N classifications, and survival time. CircRNA-MYLK was also upregulated in BC cell lines. Moreover, circRNA-MYLK promoted cell proliferation, migration, and the tube formation of HUVECs, which exhibits angiogenic potential. Mechanistically, circRNA-MYLK could directly bind to and sponge miR-29a, thus relieving suppression for target VEGFA and activating the VEGFA/VEGFR2 signaling pathway. CircRNA-MYLK is not the only circRNA that plays a critical role in tumor angiogenesis: another circRNA, circHIPK3 [119], was shown to be downregulated in Bca and suppress angiogenesis through the sponging of miR-558 and subsequent inhibition of HPSE, which could positively regulate the expression of VEGF [120, 121]. cZNF292 was also reported to be an important circular oncogenic RNA taking part in the progression of tube formation in glioma [122]. The expression of VEGF-A, EGF and active TGF-β1, as well as the levels of VEGFR-1/2, phosphorylated-VEGFR-1/2 and EGFR, were significantly downregulated following the silencing of cZNF292. Since the in-depth mechanism of cZNF292 activity is unclear, more studies are necessary.

#### Tissue invasion and metastasis

It is believed that 90% of human cancer deaths are caused by metastases and not by the primary tumor [123]. During the development of most types of human cancer, cancer cells can escape the primary tumor mass and initiate new colonies at distant sites. The process of epithelial-mesenchymal transition (EMT) has been confirmed to be essential in cell migration and tissue metastasis in cancer [124, 125]. It involves a cellular reprogramming process that drives epithelial cells into a mesenchymal-like phenotype, which is characterized by the loss of epithelial surface markers like E-cadherin and the acquisition of the mesenchymal markers vimentin and N-cadherin.

The Twist family is known as critical EMT-inducing transcription factor that increase expression of vimentin [126–128]. Meng et al. [129] demonstrated that twist1 bound to the promoter of Cul2 to activate its transcription and selectively induce expression of Cul2 circular RNA (circ-10,720) rather than mRNA. Circ-10,720 expression was high in metastatic HCC tissues and associated with clinical stage, pathology grade, metastasis and survival of patients. Circ-10,720 played an oncogenic role to promote the migration, invasion and EMT progression of HCC cells. Furthermore, it was found that twist1 promoted vimentin through increasing levels of circ-10,720, which could sponge miRNAs targeting Vimentin, including miR-1246, miR-578 and miR-490-5p. Among them, miR-490-5p was considered to be the major miRNA regulating Vimentin in HCC due to its high expression and stronger inhibitory effects to Vimentin 3′-UTR activities.

TGF-β/Smad signaling has been proven to play a crucial role in tumor metastasis and the EMT process in a variety of human cancers [130, 131]. CircPTK2 (hsa_circ_0008305) was found to be markedly downregulated in NSCLC cells during TGF-β-induced EMT [132]. Overexpression of circPTK2 arrested TGF-β-induced EMT and invasion of NSCLC cells. Mechanistically, circPTK2 functions as a sponge of miR-429/miR-200b-3p, which promotes EMT and cell invasion through targeting TIF1γ. TIF1γ is a TGF-β/Smad signaling regulator that could escalate TGF-β-induced EMT in cancer [133, 134]. In addition, circPTK2 could also negatively regulate the expression of Snail, an important downstream regulator of TGF-β/Smad signaling [135].

Compelling data revealed that SMAD2 potently contributes to EMT [136]. Zhang et al. [137] reported that circSMAD2 (hsa_circ_0000847), encoded by the SMAD2 gene, was upregulated during TGF-β-induced EMT. In addition, the expression of circSMAD2 was downregulated in HCC tissues and correlated with the tumor differentiation degree. Overexpression of circSMAD2 inhibited migration, invasion, and EMT in HCC cells through suppressing the expression of miR-629, which could promote EMT in cancer cell lines.

FOXM1 has been shown to promote cell migration, invasion and EMT in a variety of tumors [138–140]. Chen et al. [141] reported that hsa_circ_0061140 was overexpressed in ovarian cancerand could promote cell migration and invasion through regulation of the miR-370/FOXM1 pathway-mediated EMT. Hsa_circ_0061140 silencing induced a decreased expression of the EMT-related proteins, Snail and Vimentin, along with an increased expression of E-cadherin.

Circ_0067934 was shown to be upregulated in NSCLC and capable of promoting cell EMT accompained with increased N-cadherin and vimentin expression and decreased E-cadherin expression [142]. CircRNA_0023642 was upregulated in GC and acted as an oncogene by regulating EMT [143]. CircRNA_0023642 was shown to suppress expression of E-cadherin and promote expression of N-cadherin, vimentin, and snail in GC cells. Since the studies didn’t show the effector molecules of circ_0067934 and circRNA_0023642, the exact mechanisms of the two circRNAs still need to be uncovered.

In addition to the circRNAs discussed above, it is likely that many more circRNAs could regulate the hallmarks of cancer as a large number of studies have shown that circRNAs are involved in modulating proliferation, apoptosis and migration of cancer cells without exploring the underling molecular mechanisms.

### CircRNAs regulate stemness of cancer

Cancer stem cells (CSCs), a small proportion of cells that possess self-renewal and tumor-initiating capabilities, are considered to be responsible for metastatic dissemination and therapeutic failure [144–146]. Several lines of evidence have suggested that circRNAs might contribute to the stemness of cancer (Table 2).

**Table 2:** CircRNAs involved in stemness and chemotherapy resistance of cancer

Function	CircRNA	Cancer type	expression	Associated clinical features	Associated cell process	Targets	Ref
Regulating stemness	circVRK1	BC	down	–	decrease proportion of BCSCs with CD44 + CD24- phenotype, suppress BCSC’s expansion and self-renewal capacity	–	[147]
	hg19_circ_0005033	LSCC	up	–	promote proliferation, migration, invasion, and chemotherapy resistance of laryngeal cancer stem cells	miR-4521	[150]
Regulating chemotherapy resistance	circPVT1	osteosarcoma	up	enneking stage, chemoresistance, lung metastasis and survival	contributes to doxorubicin and cisplatinresistance	ABCB1	[154]
	circRNA-MTO1 (hsa-circRNA-007874)	BC	up	–	inhibit cell viability and reverse monastrol resistance	TRAF4/Eg5 axis	[157]
	circBA9.3	CML	up	–	promote resistance against TKI therapy	c-ABL1 and BCR-ABL1	[159]

Yang and colleagues [147] performed high-throughput sequencing to screen the circRNA expression profiles of breast CSCs (BCSCs) and matched non-BCSCs and found 27 aberrantly expressed circRNAs. Among these, circVRK1 was downregulated and was able to suppress the expansion and self-renewal capacity of BCSCs, displaying an inhibiting role in the stemness of BCSCs. BC cells with circVRK1 knockdown exhibited an enhanced capacity to form mammospheres and colonies, and an increasing expression of stemness-related markers including OCT4, SOX2 and NANOG, indicating that circVRK1 was involved in suppressing the stemness of BCSCs. In addition, it was speculated that circVRK1 was negatively correlated with stemness of BCSCs through the miR-153-5p/KLF5 axis, as miR-153-5p was one of the predicted miRNA targets of circVRK1 and was previously demonstrated to be involved in stemness maintenance of BC via reducing the expression of KLF5 [148]. Further investigations are necessary to support the hypothesis.

CD133 + CD44+ CSCs (TDP cells), isolated from laryngeal squamous cell carcinoma (LSCC) cells, have been shown to exhibit increased cell proliferation, migration and colony-formation ability as well as resistance to chemo- and radiotherapy [149]. These TDP cells were shown to highly express the stem-cell markers SOX2 and OCT4. In comparison with parental cells, TDP cells exhibited 3684 circRNAs by RNA sequencing (q < 0.01 and log2FC (fold change) > 1) [150]. Hg19_circ_0005033 was one of the upregulated circRNAs in TDP cells, and it could promote the proliferation, migration, invasion, and resistance to chemotherapy of TDP cells. Hg19_circ_0005033 was demonstrated to bind to miR-4521 and could function as ceRNA to upregulate miR-4521 targeted mRNAs. In addition, STAT5A, which was previously reported to induce stem-like cell properties [151], was predicted as a target of miR-4521. Thus, hg19_circ_0005033 was hypothesized to support the stem cell characteristics of CD133 + CD44+ LSCC stem cells via the miR-4521/STAT5A axis, which need further validation.

### CircRNAs regulate chemotherapy resistance of cancer

Chemotherapy represents the primary treatment for both early and advanced tumors. However, acquired resistance to chemotherapy is one of the major causes of therapeutic failure [130]. Recently, several circRNAs have been proven likely to play vital roles in the resistance of cancer to chemotherapy (Table 2).

It is well known that ATP-binding cassette B1 (ABCB1) is a multidrug resistance-related protein that is highly expressed in drug resistant cell lines and could promote resistance to chemotherapy through pumping intracellular drugs outside of the cell [152, 153]. CircPVT1 (hsa_circ_0001821), originating from exon 3 of the PVT1 gene, was significantly overexpressed in OS tissues and associated with poor prognosis of OS patients [154]. It was also upregulated in chemoresistant OS cell lines, and circPVT1 knockdown could weaken the doxorubicin and cisplatin resistance of OS cells via suppressing the expression of ABCB1.

Monastrol is a small molecule that selectively inhibits Eg5, a microtubule-based motor protein that contributes to the formation and maintenance of the bipolar mitotic spindle [155, 156]. Liu et al. [157] performed a genome-wide circRNA microarray to search for dysregulated circRNAs in the monastrol-resistant BC cells and identified circRNA-MTO1 (has-circRNA-007874) as an upregulated circRNA in these cells. Upregulation of circRNA-MTO1 promoted monastrol-induced cell cytotoxicity and reversed monastrol resistance. Mechanistically, circRNA-MTO1 could suppress expression of Eg5 through binding with TRAF4 and serve as a competing endogenous RNA (ceRNA) to repress TRAF4 from binding to the Eg5 gene.

Tyrosine kinase inhibitors (TKIs) are available for managing chronic myelogenous leukaemia (CML) [158]. Pan and colleagues [159] identified an f-circRNA, circBA9.3, generated from the BCR-ABL1 oncoprotein, that could contribute to the increased proliferation and anti-apoptotic capacities of leukaemic cells [160]. CircBA9.3 was upregulated in patients with TKI resistance and could enhance the expression of BCR-ABL1, thus contributing to resistance against TKI therapy.

### CircRNAs as biomarkers in Cancer

The properties of circRNAs mentioned in previous sections (stability, conservatism, universality, and specificity) indicate that circRNAs could be potentially valuable prognostic and diagnostic biomarkers for cancer. Recently, many studies have demonstrated circRNAs may be stably expressed and present in relatively high quantities in human body fluids, such as saliva, plasma, serum and exosomes, which also makes circRNAs ideal candidates as noninvasive liquid biopsy biomarkers for cancer [161]. Circ-ZEB1.33 was shown to be overexpressed in human HCC tissues compared to non-tumorous tissues and in serum samples from HCC patients compared to healthy controls, and its levels in HCC tissue and serum were correlated with different TNM stages and overall survival in HCC patients, suggesting circ-ZEB1.33 may serve as a valuable biomarker in HCC prognosis prediction [162]. Hsa_circ_0000190 was down-regulated in GC tissues and plasma samples [163]. Its expression levels were significantly associated with tumor size, distal metastasis, lymphatic metastasis, TNM stage and CA19–9 levels. The area under curve (AUC) of hsa_circ_0000190 in tissues and plasma were 0.75 and 0.60, respectively; the AUC of the combination was increased to 0.775, and the sensitivity and specificity of the combination were 0.712 and 0.750, respectively. In another study, hsa_circ_0000745 was shown to be lowly expressed in GC tissues and plasma samples [164]. The expression level of hsa_circ_0000745 in GC tissues was correlated with tumor differentiation, while the expression level in plasma was correlated with tumor-node metastasis stage. The AUC of hsa_circ_0000745 in plasma was 0.683, while combined with carcinoembryogenic antigen (CEA) level, the AUC increased to 0.775, suggesting good diagnostic value of hsa_circ_0000745 in plasma in combination with CEA level in GC. Zhao and colleagues [165] performed microarray screening of circRNA in saliva from oral squamous cell carcinoma patients compared with healthy controls and identified 20 downregulated and 12 upregulated circRNAs in oral squamous cell carcinoma saliva. Among these, two upregulated circRNAs, hsa_circ_0001874 and hsa_circ_0001971, showed a AUC of 0.863 and 0.845, respectively. The combination of these two circRNAs showed a AUC of 0.922. Furthermore, the risk score based on hsa_circ_0001874 and hsa_circ_0001971 could discriminate patients with OSCC from patients with oral leukoplakia with AUC for risk score 0.863, suggesting potential of salivary hsa_circ_0001874 and hsa_circ_0001971 as OSCC diagnostic biomarker. Moreover, recently studies have found that circRNAs were enriched and stable in exosomes, which are small membrane vesicles secreted by tumor cells into the extracellular fluids. Chen and colleagues [166] revealed that circPRMT5 was enriched in both serum and urine exosomes from urothelial carcinoma patients compared to healthy donors. The high levels of circPRMT5 in serum and urinary exosomes were positively associated with lymph node metastasis and advanced tumor progression, suggesting that circPRMT5 might be a prognostic biomarker in urothelial carcinoma. In addition, it was found that other circRNAs, such as hsa_circ_0006633 [167], hsa_circ_0000520 [168], hsa_circ_0000673 [169], hsa_circ_0001017 [170], hsa_circ_0061276 [170], circ-TTC17 [171], circ-LDLRAD3 [172], hsa_circ_0001785 [173], hsa_circ_0001445 [174], hsa_circ_0000181 [175], hsa_circ_0013958 [176] and hsa_circ_0000285 [177, 178], were also detectable in plasma, serum or exosomes and could distinguish patients with cancer from healthy controls and were potential valuable biomarkers in cancer (Table 3).

**Table 3:** CircRNAs as liquid biopsy biomarkers in cancer

Source	Cancer type	Cohort size	CircRNA	Expression	Associated clinical features	Ref
saliva	OSCC	90 OSCC patients, 70 OLK subjects	hsa_circ_0001874	up	TNM stage, tumor grade	[165]
	OSCC	90 OSCC patients, 70 OLK subjects	hsa_circ_0001971	up	TNM stage	[165]
plasma	GC	104 GC patients, 104 healthy individuals	Hsa_circ_0000190	down	tumor diameter, lymphatic metastasis, distal metastasis, TNM stage, CA19–9levels	[163]
	GC	20 GC patients, 20 healthy individuals	hsa_circ_0006633	up	distal metastasis, tissue carcinoembryonic antigen level	[167]
	GC	45 GC patients, 17 healthy individuals	hsa_circ_0000520	down	CEA expression	[168]
	GC	24 GC patients, 14 healthy individuals	hsa_circ_0000673	down	TNM stage	[169]
	GC	121 GC patients, 121 healthy individuals	hsa_circ_0001017, hsa_circ_0061276	down	OS, DFS	[170]
	ESCC	30 ESCC patients, 25 healthy individuals	Circ-TTC17	up	TNM stage, lymphatic metastasis, OS	[171]
	PC	31 PC patients, 31 healthy individuals	circ-LDLRAD3	up	CA19–9, N classification, venous invasion, lymphatic invasion	[172]
	BC	57 BC patients, 17 healthy individuals	hsa_circ_0001785	down	histological grade, TNM stage, distant metastasis	[173]
	HCC	104 HCC patients, 52 healthy individuals	hsa_circ_0001445	down	AFP level	[174]
	GC	102 GC patients, 105 healthy individuals	Hsa_circ_0000181	down	tumor differentiation, carcinoembryonic antigen	[175]
	LAC	30 LAC patients, 30 healthy individuals	hsa_circ_0013958	up	TNM stage, lymphatic metastasis	[176]
serum	HCC	64 HCC patients, 30 healthy individuals	circ-ZEB1.33	up	TMN stages, OS	[162]
	BCa	197 BCa patients, 97 healthy individuals	hsa_circ_0000285	down	tumor size, differentiation, lymph node metastasis, distant metastasis, TNM stage, OS	[177]
	NPC	150 NPC patients, 100 healthy individuals	circRNA_0000285	up	tumor size, differentiation, lymph node metastasis, distant metastasis, TNM stage.	[178]
exosome (serum and urine)	UCB	71 UCB patients, 36 healthy individuals	circPRMT5	up	lymph node metastasis, T and N status, DFS	[166]
	UCB	18 UCB patients, 14 healthy individuals	circPRMT5	up	lymph node metastasis, T and N status, DFS	[166]

## Conclusions

CircRNAs were previously thought to represent errors during the process of RNA splicing. Fortunately, in the past few years, accumulating evidence has illustrated the significant regulatory effects of circRNAs on pathophysiologic processes, including tumorigenesis. CircRNAs are now regarded as a class of abundant, stable, diverse and conserved RNA molecules with a range of activities, including sponge, translation, splicing and regulation. The functions of circRNAs in cancer are gaining considerable interest and have become a focus of cancer research. In this review, we briefly summarized the recent advances regarding circRNAs in the hallmarks, stemness, resistance to therapy, and the possibility as biomarkers for cancer.

These research endeavors into circRNAs expand our understanding of eukaryotic transcription participants and their important roles in organisms, especially in cancer. The stability, conservatism, universality, and specificity of circRNAs make it to be a potential valuable prognostic and diagnostic biomarker for cancer, and the functions and regulatory roles that circRNAs play in tumor cells make it possible to be a target for the treatment of cancer. However, the study of circRNAs in cancer remains in its infancy. CircRNAs are far from being able to be incorporated into clinical practice, and there are still fundamental problems necessitating further investigation in this field. For example, there is an urgent need to develop a common standardized naming system for circRNA research. In addition, further investigation is needed regarding the precise mechanisms, other than those of miRNA sponge activity, of circRNAs underlying the initiation and progression of cancer. Furthermore, more controlled and large-scale clinical studies are required before cancer-specific circRNAs can be recommended for diagnosis and treatment. An advanced understanding of circRNA will provide beneficial insights and generate new hypotheses regarding cancer pathogenesis. We hope that the appropriate and precise use of circRNAs in clinical applications might eventually create breakthroughs for cancer therapy in the near future.

## Abbreviations

3′-UTR: 3′-untranslated region
ABCB1: ATP-binding cassette B1
AKT: Protein kinase B
As-HaCaT: Arsenite-treated HaCaT
AUC: Area under curve
BAX: BCL2-associated X Protein
BC: Breast cancer
Bca: Bladder cancer
bcl-2: B-cell lymphoma-2
BCSCs: Breast CSCs
CCNE1: Cyclin E1
CDK: Cyclin-dependent kinase
CDR1as: Cerebellar degeneration-related protein 1 antisense RNA
ceRNA: Competing endogenous RNA
circ-Amotl1: CircRNA derived from angiomotin-like1
CircRNAs: Circular RNAs
ciRNA: Circular intronic RNA
CML: Chronic myelogenous leukaemia
CRC: Colorectal cancer
CSCs: Cancer stem cells
DNMT1: DNA methyltransferase 1
DOCK1: Dedicator of cytokinesis 1
ecircRNA: Exonic circRNA
ECM: Extracellular matrix
EGFR: Epidermal growth factor receptor
EIciRNA: Exon-intron circRNA
EIF3J: Eukaryotic translation initiation factor 3 subunit J
EMT: Epithelial-mesenchymal transition
EZH2: Enhancer of zeste homolog 2
FLI1: Friend leukemia virus integration 1
Foxo4: Forkhead Box O 4
GC: Gastric cancer
HPSE: Heparanase
HuR: Human antigen R/ELAV-like protein 1
IGF1R: Insulin-like growth factor I receptor
IRES: Internal ribosome entry site
ITGB8: Integrin subunit beta 8
LSCC: Laryngeal squamous cell carcinoma
MREs: miRNA response elements
MYLK: Myosin Light Chain Kinase
NSCLC: Non-small cell lung cancer
nt: Nucleotide
ORF: Open reading frame
OS: Osteosarcoma
PAIP2: Polyadenylate-binding protein-interacting protein 2
PD-L1: Programmed death-ligand 1
PIK3CD: Phosphatidylinositol-4,5-bisphosphonate 3-kinase, catalytic subunit delta gene
PRC2: Polycomb-repressive complex 2
PTEN: Phosphatase and tensin homolog deleted on chromosome ten
ROCK1: Rho-associated protein kinase 1
Smad: Mothers against decapentaplegic
snRNPs: Small nuclear ribonucleic proteins
TDP cells: CD133 + CD44+ CSCs
TERT: Telomerase reverse transcriptase
TGF-β: Transforming growth factor-β
TIF1γ: Transcriptional intermediary factor 1 γ
TKI: Tyrosine kinase inhibitor
VEGF: Vascular endothelial growth factor
VEGFR: Vascular endothelial growth factor receptor
YAP1: Yes-associated protein 1
YY1: Yin Yang-1
